#  New Concepts in Pacemaker Syndrome

**Published:** 2004-10-01

**Authors:** D. Michael Farmer, NA. Mark Estes, Mark S Link

**Affiliations:** Tufts-New England Medical Center, Tufts University School of Medicine, Boston, Massachusetts

**Keywords:** VVI = ventricular-based, VVIR = rate modulated ventricular-based, A-V = atrioventricular, V-V = interventricular, V-A = ventricular-atrial, LBBB = left bundle branch block, LVEF = left ventricular ejection fraction, DDDR = rate modulated dual-chamber, SND = sinus node dysfunction, AAI = single-chamber atrial, AF = atrial fibrillation, LV = left ventricular, RV= right ventricular

## Abstract

After implantation of a permanent pacemaker, patients may experience severe symptoms of dyspnea, palpitations, malaise, and syncope resulting from pacemaker syndrome.  Although pacemaker syndrome is most often ascribed to the loss of atrioventricular (A-V) synchrony, more recent data may also implicate left ventricular dysynchrony caused by right ventricular pacing.  Previous studies have not shown reductions in mortality or stroke with rate-modulated dual-chamber (DDDR) pacing as compared to ventricular-based (VVI) pacing. The benefits in A-V sequential pacing with the DDDR mode are likely mitigated by the interventricular (V-V) dysynchrony imposed by the high percentage of ventricular pacing commonly seen in the DDDR mode.  Programming DDDR pacemakers to encourage intrinsic A-V conduction and reduce right ventricular pacing will likely decrease heart failure and pacemaker syndrome.  Studies are currently ongoing to address these questions.

## Introduction

Pacemaker syndrome consists of the cardiovascular signs and symptoms of heart failure and hypotension induced by right ventricular (RV) pacing.  The reported incidence of pacemaker syndrome likely approaches 20% in rate modulated ventricular-based (VVIR) pacing [1]. Over the last three decades the understanding of pacemaker syndrome has evolved.  Initially described as only the sequela of A-V dysynchrony, pacemaker syndrome may be highly influenced by RV-left ventricular (LV) dysynchrony as well.  The intricate interplay of these two factors along with autonomic and neurohormonal changes cause the compilation of symptoms known as the pacemaker syndrome.

## Pacemaker Syndrome

Pacemaker syndrome was first described in 1969 by Mitsui et al. as a collection of symptoms associated with right ventricular pacing [2].   Since its first discovery, there have been many definitions of pacemaker syndrome, and the understanding of the cause of pacemaker syndrome is still under investigation.  In a general sense, pacemaker syndrome can be defined as the symptoms associated with right ventricular pacing relieved with the return of A-V and V-V synchrony.

The symptoms of pacemaker syndrome included dyspnea on exertion, paroxysmal nocturnal dyspnea, orthopnea, hypotension, pre-syncope, and even syncope [3-5].  Heart failure signs include elevated neck veins, rales, and pedal edema.  Physical exam can often reveal cannon A-waves.  This sign occurs secondary to ventricular-atrial (V-A) conduction and the contraction of the atria against closed A-V valves.  Although relatively uncommon, syncope has been attributed to pacemaker syndrome.  Syncope is usually associated with systolic blood pressure declines of greater than 20 mm Hg that can occur with the onset of pacing.  Additional symptoms attributed to pacemaker syndrome include easy fatigability, malaise, headache, and the sensation of fullness and pulsations in the head and neck.  Pacemaker syndrome is most severe when intact V-A conduction is present6.  The elevated venous pressures associated with the contraction against closed A-V valves causes a vagal afferent response resulting in peripheral vasodilation and hypotension.

## Incidence

The reported incidence of pacemaker syndrome has ranged from 2% [7] to 83% [8].  The wide range of reported incidence is likely attributable to two factors.  The first is the criteria used to define pacemaker syndrome.  In the Pacemaker Selection in the Elderly (PASE) study, pacemaker syndrome was defined as symptoms severe enough to warrant reprogramming from ventricular to dual-chamber pacing [9].   The Mode Selection Trial (MOST) investigators defined pacemaker syndrome as occurring if either one of two different criteria occurred [1].  The first criteria was new or worsened dyspnea, orthopnea, elevated jugular venous pressure, rales, and edema with ventricular (VA) conduction during ventricular pacing.  The second criteria was symptoms of dizziness, weakness, presyncope, or syncope, and a >20 mm Hg reduction of systolic blood pressure when the patient had VVIR pacing compared with atrial pacing or sinus rhythm.   The second factor in the wide range of reported incidence of pacemaker syndrome is the therapy used to resolve that diagnosis.   When surgical revision is required to upgrade a patient from VVIR pacing, the incidence of pacemaker syndrome has been low.  In the Canadian Trial of Physiologic Pacing (CTTOP), surgical revision was required for the change from VVIR to dual chamber pacing, and the incidence of pacemaker syndrome was reported to be 2.7% at three years [10].  In other pacemaker mode trials, patients were implanted with dual chamber devices and then either programmed to VVIR or DDDR pacing.  In these studies, patients complaining of symptoms consistent with pacemaker syndrome could be easily upgraded to DDDR mode by simple pacemaker reprogramming.  In the PASE and MOST studies  in which devices could be reprogrammed from VVIR to DDDR mode,  the incidence of pacemaker syndrome was higher than in those studies that required an invasive intervention to change pacing mode [9,11].

## A-V vs. V-V dysynchrony

The majority of the symptoms of pacemaker syndrome are likely attributable to the reduction in cardiac output that is associated with right ventricular pacing [3-5].  Several studies have demonstrated the hemodynamic superiority and increase in cardiac output of A-V sequential pacing over ventricular pacing [12].  Other studies have shown that A-V and V-V synchrony are independent contributors to the hemodynamic ramifications of right ventricular pacing [13,14].  Right ventricular pacing with or without A-V synchrony induces a physiologic contraction similar to that caused by left bundle branch block (LBBB).  The effects of LBBB have been well studied.  LBBB leads to an asynchronous ventricular contraction leading to altered diastolic filling time, increase in mitral regurgitation, as well as a reduction in left ventricular ejection fraction (LVEF) [15-17].  Thus, the reduction in cardiac output and symptoms associated with pacemaker syndrome are likely secondary to the loss of both A-V and V-V synchrony that is associated with right ventricular apical pacing [18].

##  “Physiologic” vs. Ventricular pacing Trials

The physiologic benefits of A-V sequential have caused DDDR pacing to become common practice in most patients with sinus node dysfunction (SND).  The results of completed randomized clinical trials of pacemaker mode selection have been somewhat conflicting.   Overall, most trials have not shown reduction in heart failure, reduction in mortality, or improvement in quality of life with A-V sequential pacing (Table 1).

In the first of such trials, Anderson et al. compared single-chamber atrial (AAI) with VVI pacing in 225 patients with SND, normal A-V conduction, and a narrow QRS who had standard pacing indications [7].  The primary endpoints were frequency of atrial fibrillation (AF) and thromboembolic events.  The original study follow-up was 3.3 years with a subsequent analysis at 5.5 years.  Long term follow-up demonstrated persistent reduction in the primary endpoints of AF, thromboembolic events, chronic AF, and all cause mortality in the AAI paced group.  At longer follow-up, the VVI group had an increased incidence of heart failure, worsening echocardiographic measurements of LV function, and increase in all-cause mortality.  These long-term results have not been reproduced by any other prospective, randomized trial comparing solely atrial-based to ventricular-based pacing, although the pacing mode in subsequent trials was mainly DDDR.

In the PASE study, 407 patients older than 65 years of age, in sinus rhythm, who required a pacemaker for bradycardia, were randomized to VVIR or DDDR pacing [9].  The primary end point was quality-of life.  Secondary endpoints included death from all causes, first nonfatal stroke or death, first hospitalization for heart failure, development of atrial fibrillation, and the development of pacemaker syndrome.  At quality of life evaluations at 3, 9 and 18 months, there were no differences between the two pacing modes.  There was a 26% crossover rate due to the development of pacemaker syndrome. No differences in clinical outcomes were observed.  There were some trends that showed benefit in clinical outcomes in the DDDR group, especially in the subset of patients with SND, but none were of statistical significance.

As a result of the conflicting data in the Anderson et al. trial and the PASE study, two larger trials in pacemaker mode selection were performed.  In the Canadian Trial of Physiologic Pacing (CTOPP) trial, 2568 patients with symptomatic bradycardia requiring pacing were randomized to atrial-based (AAI, AAIR, DDD, or DDDR) or ventricular pacing (VVI or VVIR) [10].  AAI and AAIR combined for only 5% of the patients assigned to the atrial-based group.  The remaining 95% in the atrial-based group were either DDD or DDDR paced.  The combined endpoint of stroke or death due to cardiovascular causes after a 3-year follow-up was not different between the two groups.  The secondary endpoints of AF and chronic AF were observed less commonly in the atrial-based group.  After an eight year follow-up, there were no significant differences in death or stroke between the two groups [19].

In the Mode Selection Trial in Sinus Node Dysfunction (MOST) study, 2020 patients with SND received dual chamber pacemakers and were randomized to either VVIR or DDDR pacing [11]. The mean follow-up was 33 months and the primary endpoints were death and nonfatal stroke.  There were no differences in the primary endpoint between the two groups.  The incidence of atrial fibrillation was lower with dual-chamber pacing.  Subsequent analyses of the MOST data, has shown that the benefits of A-V sequential pacing are likely attenuated by the ventricular pacing that occurs in the DDDR mode [20]. Patients with a pre-paced QRS duration less than 120 ms were analyzed for percentage of ventricular pacing and clinical events.  The percentage of ventricular pacing was determined from stored pacemaker data. The percentage of ventricular pacing was greater in the DDDR versus VVIR mode (90% vs. 58%).  The percentage of ventricular pacing was a strong predictor of heart failure hospitalization in both pacing modes.  The risk of AF increased linearly with percentage of ventricular pacing in both groups.  The authors concluded that ventricular desynchronization imposed by ventricular pacing even when A-V synchrony is preserved increases the risk of heart failure hospitalization and AF in SND with normal baseline QRS duration.  The analyses by Sweeney et al. illustrate the fact that DDDR and DDD pacing is not physiologic.  The pacing mode that is most physiologic is AAI or AAIR. This fact can likely explain the discrepancies between the study by Anderson et al. and the subsequent studies in pacemaker mode selection.  The benefit derived from A-V sequential pacing in the CTTOP and MOST studies was likely counterbalanced by the detrimental effects of right ventricular pacing seen in the DDDR mode.

Several ongoing trials are looking at the benefits of reduction in RV pacing in the DDDR mode. The first is the Danish Multicenter Randomized Study of Atrial Inhibited Versus Dual-Chamber Pacing in Sick Sinus Syndrome (DANPACE).  This trial is comparing AAI pacing and DDDR pacing with a short A-V delay.  The second is the Search A-V Extension for Promoting Atrioventricular Conduction (SAVE-PACE) study.  This study will evaluate the use of search hysteresis, a pacemaker feature that allows the extension of the A-V delay to reduce right ventricular pacing.  The study investigators hypothesize that the reduction in RV pacing will lead to a reduction in left ventricular remodeling and AF.

##  Conclusion

Pacemaker syndrome is a common problem faced by clinicians who implant pacemakers and for those who take care of these patients.  The syndrome is likely caused by both the loss of A-V and V-V synchrony imposed by right ventricular pacing.  The studies on pacemaker mode selection have shown a high incidence of pacemaker syndrome.  Because of the high incidence of pacemaker syndrome in VVIR-paced patients, atrial-based pacing is preferred.

These studies have also showed that DDDR pacing as compared to VVIR pacing decreases the incidence of AF, but does not affect stroke or mortality.  The percentage of beats ventricular paced in these studies in patients who received dual-chamber pacemakers is likely to influence the incidence of pacemaker syndrome and heart failure.  The amount of ventricular pacing in the DDDR mode is dependent on spontaneous A-V conduction and programmed A-V delay.  Optimal programming should seek to reduce ventricular pacing.  The reduction in the percentage of RV pacing in dual-chamber pacing modes will likely reduce the incidence of pacemaker syndrome, heart failure, and possibly mortality.

## Figures and Tables

**Table 1:**
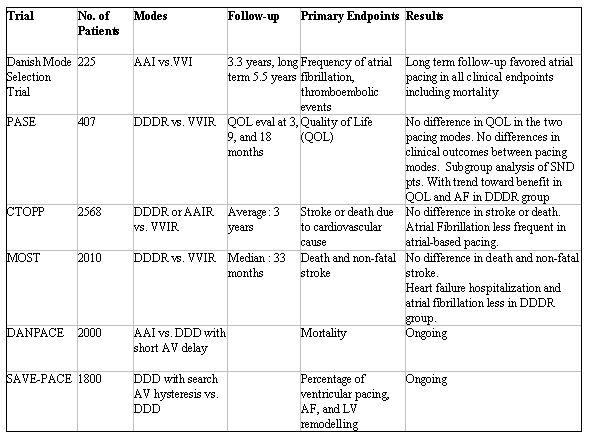
Clinical Trials of Pacemaker Mode Selection
